# Systematic Analysis and Prediction of Pupylation Sites in Prokaryotic Proteins

**DOI:** 10.1371/journal.pone.0074002

**Published:** 2013-09-03

**Authors:** Xiang Chen, Jian-Ding Qiu, Shao-Ping Shi, Sheng-Bao Suo, Ru-Ping Liang

**Affiliations:** 1 Department of Chemistry, Nanchang University, Nanchang, P.R. China; 2 Department of Materials and Chemical Engineering, Pingxiang College, Pingxiang, P.R. China; 3 Department of Mathematics, Nanchang University, Nanchang, P.R. China; Semmelweis University, Hungary

## Abstract

Prokaryotic ubiquitin-like protein (Pup) is the first identified prokaryotic protein that is functionally analogous to ubiquitin. Recent studies have shed light on the Pup activation and conjugation to target proteins to be a signal for the selective degradation proteins in *Mycobacterium tuberculosis* (Mtb). By covalently conjugating the Pup, pupylation functions as a critical post-translational modification (PTM) conserved in actinomycetes. Detecting pupylation sites is crucial and fundamental for understanding the molecular mechanisms of Pup. Yet comparative studies with other PTM suggest that the development of accurate and complete repertories of pupylation is still in its early stages. Unbiased screening for pupylation sites by experimental methods is time consuming and expensive; *in silico* prediction can provide highly potential candidates and reduce the number of potential candidates that require further *in vivo* or *in vitro* confirmation. Here, we present an effective classifier of PupPred for predicting pupylation sites, which shows better performance than existing classifiers. Importantly, this work not only investigates the sequential, structural and evolutionary hallmarks around pupylation sites but also compares the differences of pupylation and ubiquitylation from the environmental, conservative and functional characterization of substrates. These prediction and analysis results may be helpful for further experimental investigation of degradation proteins in prokaryotes. Finally, the PupPred server is available at http://bioinfo.ncu.edu.cn/PupPred.aspx.

## Introduction

Cellular pathways involved in determining the fate of essential proteins through post-translational modification events have become an increasingly important area of study [1]–[4]. Of these modifications, the understanding of eukaryotic ubiquitylation by ubiquitin protein has shown to be especially valuable [5]. With the ability to mark specific proteins for proteasomal degradation, this pathway has been shown to play a particular important role in the cell cycle, cellular metabolism, cell signaling and immune response [6]–[8]. Similar to eukaryotic ubiquitin, prokaryotic ubiquitin-like protein (Pup) attaches to specific lysine residues of substrate proteins by forming isopeptide bonds to target the proteins for proteasomal degradation in prokaryotes [2]–[4], [9]. This pathway also has been shown to play a particular important role for proteasomal degradation in the prokaryotes [4], [9]. While the eukaryotic ubiquitin–proteasome degradation pathway was discovered in the late 1970’s [10], it was only recently that an ubiquitin-like protein was identified in prokaryotes [4], [7]. This new Pup has now been characterized from the Actinobacterium *Mycobacterium tuberculosis* (Mtb) and its avirulent relative *Mycobacterium smegmatis* (Msm). Since the proteasomal pathway is critical for both the virulence and persistence of Mtb, identification of the pupylated substrates along with information on the exact sites is fundamental for understanding the pathological mechanisms, and can provide helpful insights into protein degradation in actinomycetes.

Recently, large-scale proteomics technology has been applied to identify pupylated proteins and pupylation sites [11]–[14]. However, experimental determination of pupylated substrates with exact modified sites is still a great challenge, and no canonical sequence motifs have been observed [12], [13]. In contrast to labor-intensive and time-consuming experimental approaches, the *in silico* prediction of putative pupylation sites with high predictive performance can greatly narrow down the number of potential targets, and thus rapidly provide useful information for further experimental confirmation. In this regard, an accurate and convenient predictor of pupylation is urgently needed.

Up to now, only one method for the prediction of pupylation sites was constructed based on a GPS algorithm, in which three sequential steps of motif length selection (MLS), weight training (WT) and matrix mutation (MaM) were adopted [15]. It was trained on a combined set that manually collected 127 experimentally identified pupylation sites in 109 prokaryotic proteins from the scientific literature. Although the method demonstrated a promising accuracy, it is nevertheless still a shortage of low sensitivity. Moreover, no published reports including the above method have systematically studied not only the biological hallmarks for pupylated substrates but also the differences of pathway for pupylation and ubiquitylation. Based on these factors, we present a new computational tool known as PupPred, which was constructed to predict the pupylation of prokaryotic proteins by using the latest data of PupDB database [16]. The database PupDB contains 182 pupylated proteins with 215 known pupylation sites (see Table S1). In this work, we found the composition of amino acid pairs is suitable for representing the sequence context surrounding the pupylation sites after our preliminary assessment by seeking a various encoding. The PupPred achieved a balanced performance of both high sensitivity and specificity by using the encoding scheme of amino acid pairs based on a more training data, and that outperformed the GPS-based predictor when evaluated on the same dataset. More importantly, the sequential, structural and evolutionary hallmarks around pupylation sites were exhibited. Previously, we had developed a tool known as UbiProber for the prediction of eukaryotic ubiquitylation sites [17]. Since ubiquitylation and pupylation are functional analogues, we tried to use the method of UbiProber to predict prokaryotic pupylation sites. Unfortunately, a poor prediction results were obtained (see Table S2), where may be partially due to different sequence functionality and constraints between ubiquitylation and pupylation. So, we systematically compared pupylation with ubiquitylation through comparing the differences of the environmental, conservative hallmarks and statistical analyzing of their respective gene ontology (GO) terms. Taken together, these systematic analyses and predictions can allow us to gain better insights into processes and functions of pupylation.

## Results

### Sequence-derived Hallmarks of Pupylation Sites

This investigation focuses on the analysis of ubiquitin-like protein conjugated lysine in prokaryotes. In ubiquitin-like protein conjugation, the region of the ubiquitin-like protein conjugated lysine residues is in directly contact with the proteasome accessory factor A (PafA) catalytic center [18], [19]. Since PafA has a substrate binding specificity, whether the region of ubiquitin-like protein conjugated lysine conservative amino acid motifs for PafA recognition must be explored. After the duplicated sequences of experimental pupylation sites were removed, as shown in Figure S1, a web-based tool TwoSampleLogo [20] was adapted to generate the graphical sequence logo (*P*<0.01; *t*-test) that detects and displays statistically significant differences in position-specific symbol compositions between two sets of multiple sequence alignments. One interesting feature is the absence of additional lysines at positions that are immediately adjacent to the pupylation site. For example, lysines are depleted at positions −2 and 2 (see Figure S1). This suggests that pupylation sites do not have a tendency to cluster, perhaps due to the structural constrains that would prevent simultaneous attachment of two or more bulky ubiquitin-like protein molecules in close proximity to each other on the same substrate. Another interesting feature is the upstream and adjacent amino acid residues in pupylated sites, which may be close to pupylated lysine residues in three-dimensional structure, have notable difference between pupylation sites and non-pupylation sites.

**Figure 1:**
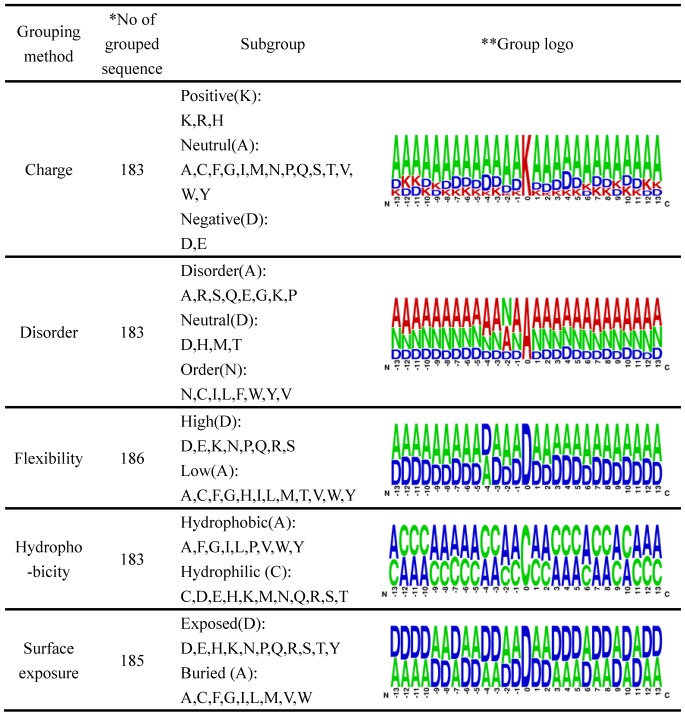
The graphical representation of biochemical environment surrounding pupylation sites using different grouping method. *Redundant sequence is eliminated from each group sequence; **each symbol in the stack of group logo represents each class in the group column.

In addition, since the representation of sequence logos involves different preferences of amino acids for pupylated and non-pupylated sites, the statistical difference in the distribution of amino acid sequences around the pupylated lysines can be alternatively grouped by various methods to generalize the sequence hallmarks because amino acid classification is hierarchical. Here, WebLogo [21] was adapted to generate the graphical sequence logo for the sequence hallmarks at each position around the pupylated sites. The sequence hallmarks around the pupylation sites can then be easily investigated. As presented in Figure 1, the three-class grouping method and the two-class grouping method are used to divide 20 amino acids into subgroups that capture their chemical properties. Three-class grouping methods can be based on charge and disorder, and two-class grouping methods can be included flexibility, hydrophobicity and surface exposure. The majority of pupylated sites are located in the uncharged, unstructured (disorder) and high flexibility regions. Moreover, the result also shows a slight tendency to prefer hydrophobic and exposed regions.

### Structural Hallmarks of Pupylation Sites

**Figure 2:**
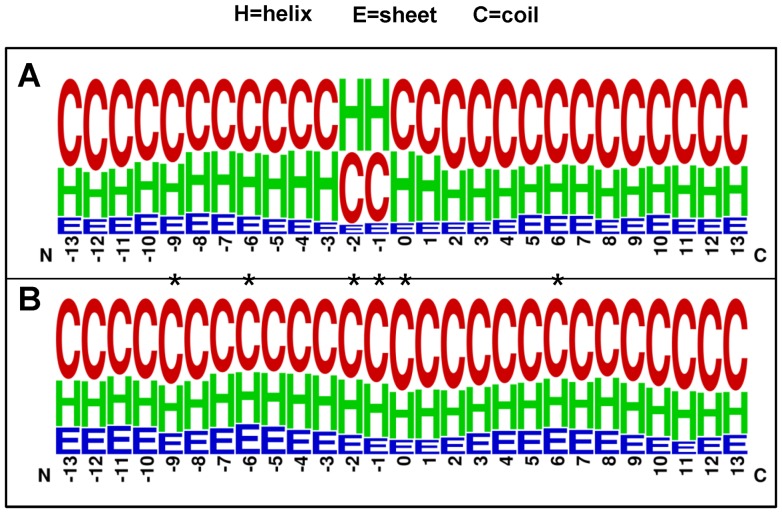
A weblogo of secondary structure of pupylated sequences (A) and non-pupylated sequences (B). *P*-values were calculated using the Welch’s *t*-test (two tails). *The compositional biases of secondary structure are statistically significance (*P*<0.001) comparing pupylated sequences with non-pupylated sequences.

Besides composition of amino acids, we further analyze the correlation of structural information at pupylation sites. Since most of the experimentally verified pupylated proteins do not have corresponding protein tertiary structures in Protein Data Bank (PDB) [22]. Then, PSIPRED [23], a highly accurate method for protein secondary structure prediction, was applied to compute the secondary structure of each residue in the protein sequence. Figure 2 presents the sequence logo of the secondary structure in the 27-mer window (−13∼+13) of the pupylated and non-pupylated sites. We also calculated statistically significant differences in the distribution of secondary structure based on the paired Welch’s *t*-test. In this work, the observations reveal that PafA prefers to recognize the regions that are located in the middle of coil and helix structures (*P*<0.02017; see Table S3). In contrast to pupylation sites, non-pupylation sites do not have an obviously preferred secondary structure.

To gain better insights into structural preferences of pupylated sites, we searched the available structural information for proteins from our positive sample (182 pupylated proteins) using BLAST against the PDB with ≥70% sequence identity as a cutoff value. Our search resulted in a total of 79 homologous protein chains containing 43 pupylated proteins that the exact structural information is known (see Table S4). There are 55 pupylated sites in the 43 pupylated protein. Despite the presence of more than 80,000 structures in PDB, reliable structural assignments can be made for only ∼26% of the available pupylated sites (55 out of 215 pupylated sites). This indicates that very limited structural environment information is currently available for proteins that comprise known pupylated substrates. Interestingly, our analysis showed that the 55 sites could be confidently assigned to ordered regions, and 10 were located within coils, 23 within helices, and 22 within strands. The majority of the sites within helices and coils were surface exposed and had high B-factor values indicating high flexibility.

### Evolutionary Hallmarks of Pupylation Sites

**Figure 3:**
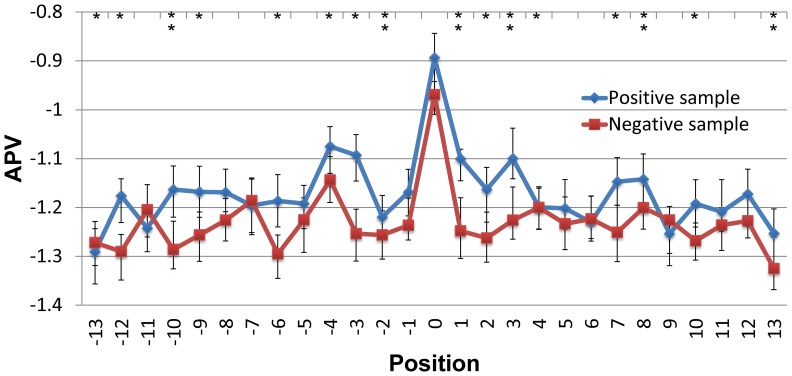
Average PSSM value of amino acids for each position of pupylated sequences and non-pupylated sequences. *P*-values were calculated using the Welch’s *t*-test (two tails). **P<0.0001; *P<0.001.

To compare evolutionary information for each protein sequence, the corresponding position-specific scoring matrix (PSSM) was obtained by applying PSI-BLAST [24] search against Swiss-prot database. Figure 3 presents the comparison of average PSSM value (APV) between pupylated and non-pupylated sequences. *P*-values also were calculated using the Welch’s *t*-test (two tails) for each position in the windows, as shown in Table S3. This analysis showed a high preference for conservative in pupylation sites. Moreover, the amino acid residues adjacent to the centered pupylation sites have relatively higher preference (*P*<8.234e-04; see Table S3) for conservative than those of non-pupylation sites, especially in the region of downstream sequences (+1, +2, +3). Also particular, the upstream positions of pupylation sites (−12, −10, −9, −6, −3) have a high score, where are significantly conservative for pupylation sites (*P*<6.024e-04; see Table S3).

### Predictive Performance of Cross-validation using Various Training Features

**Figure 4:**
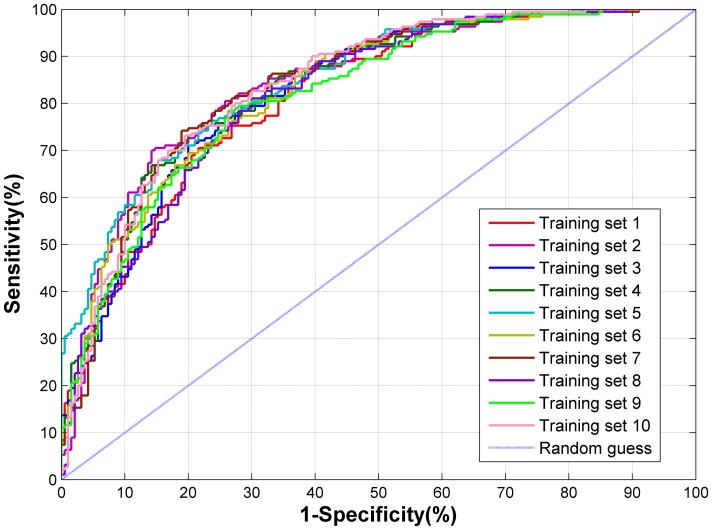
ROC curves of PupPred predictions on 10 training sets.

As mentioned earlier, pupylation and non-pupylation have some significant differences in sequence, structural and evolutionary hallmarks. Therefore, we constructed models that differentiate between pupylation sites and non-pupylation sites from these three aspects. Here, to determine which features can be utilized to construct model, we preliminarily assessed various features by *k*-fold cross validation that including binary encoding, amino acid (AA) compositions, AA pair compositions, grouping amino acid composition, physicochemical properties, *k* nearest neighbor (KNN) feature, secondary structure and position specific scoring matrix (PSSM) profile. Table 1 presents the predictive performance achieved using various training features, based on 10-fold cross-validation for each one of 10 training sets. Of the models trained using individual features, those that were trained using AA pair composition slightly outperformed those that were trained using other features. In addition, the model trained with the PSSM profile, KNN feature or binary encoding achieved a Matthew correlation coefficient (*MCC*) of over 0.2. However, the model that was trained with the secondary structure or AA composition underperformed prediction. According to the prediction performance of the individual features, we selected three features with high dimension and powerful predictive ability for further optimization by using a feature selection method known as F-select [25]. Therefore, the effects of binary encoding, PSSM profile and AA pair composition were evaluated, as presented in Table 1. According to statistical comparison of *MCC* (see Table S5) and the evaluation results (see Table 1), the model was trained using the 121 informative features (Table S6) of AA pair composition performed best (*P*<2.797e-05), with the best-balanced predictive sensitivity and specificity. AA pair composition is suitable for representing the sequence context surrounding the pupylation sites because AA pair composition reflects the short range interactions of residues within a sequence or a sequence fragment. Note that all evaluation criteria come from the average of the prediction results of 10 training sets. The detailed evaluation results of 121 informative features for these 10 training sets are listed in Table S7. Furthermore, the receiver operating characteristic (ROC) curves are drawn in Figure 4 and the corresponding value of average area under the curve (AUC) was 83.26%. Since the prediction performance of different training sets is extremely stable for the prediction of pupylation sites, it is evident that the method is a robust predictor. In short, the performance of our model is reasonably good.

**Table 1:** The predictive performance of cross-validation using various training features.

Training features	Sn (%)	Sp (%)	Ac (%)	*MCC*
Binary encoding	43.36±5.00*	75.80±4.25	59.58±3.56	0.2028±0.0744
AA composition	64.14±4.00	52.79±2.94	58.46±3.14	0.1708±0.0635
AA pair composition	62.46±6.88	62.48±4.37	62.47±4.29	0.2503±0.0861
Grouping AA composition	41.78±5.33	76.04±3.70	58.91±3.51	0.1902±0.0741
Physicochemical properties	55.53±4.96	63.93±4.54	59.73±2.84	0.1960±0.0571
KNN feature	64.94±5.68	55.85±6.74	60.39±2.46	0.2105±0.0490
Secondary structure	59.96±2.48	57.40±6.11	58.68±2.14	0.1739±0.0433
PSSM	51.20±4.36	69.39±6.19	60.30±2.49	0.2110±0.0518
Binary encoding_233_ **	64.04±4.33	78.60±1.36	71.63±2.03	0.4320±0.0414
PSSM_134_	61.11±2.63	68.94±1.28	65.11±2.13	0.3019±0.0331
AA pair composition_121_	**76.24±1.25**	**75.32±0.96**	**75.78±1.07**	**0.5191±0.0215**

* The latter number represents standard deviation from 10 training set;

** the lower right corner of the number indicates the number of selected best feature sets by the F-score method.

### PupPred Server

After evaluating the trained models for identifying pupylation sites, the model with the highest predictive accuracy for 10 training sets was selected as the PupPred server. PupPred web server has been developed in an easy-to-use manner and is available to the general public. A user can visit PupPred at http://bioinfo.ncu.edu.cn/PupPred.aspx, input the protein sequences in FASTA format into the text box, and run the program by clicking the ‘Predict with the pasted sequence’ linkbutton. Moreover, users can paste the protein IDs into the text box, and run the program by clicking the ‘Predict with the protein ID’ linkbutton. The prediction results should be regarded as potential sites before experimental validation.

### Comparison of PupPred with Existing Methods

We attempted to compare the PupPred with the GPS-PUP. However, the GPS-PUP adopted the unbalanced ratio of positive to negative samples to train model, which resulted in a comparatively unjust assessment between these two predictors. Unbalanced datasets present a number of different problems for machine learning methods [26]. When only a comparatively small number of examples are available for one class, the machine learning algorithm will not have sufficient information to learn a function to distinguish the classes. Reporting of classification accuracy is also impacted by unbalanced datasets [27], [28]. For example, if a dataset of 100 sites contains 20 pupylation sites and 80 non-pupylation sites, a classification accuracy of 80% can be obtained by classifying all sites as negative.

To demonstrate the problem, we designed an experiment to investigate the effect of unbalanced datasets on pupylation prediction. For this experiment, there are many more non-pupylation sites than pupylation sites available. Two different approaches were used to build classifiers for comparing the GPS-PUP: (1) the prediction model was constructed by using balanced training set (PupPred, the classifier of this research); (2) the model was constructed by using unbalanced training set (PupPred^1^, the original ratio of positive and negative samples). To compare the performance, we submitted the testing data of 109 complete protein sequences to the classifiers (PupPred, PupPred^1^ and GPS-PUP). The evaluation results show that the PupPred^1^ and GPS-PUP predicted almost all examples to be negative (i.e. low sensitivity and high specificity). In contrast, PupPred trained by using balanced data had higher classification accuracies at three stringency levels of high, medium and low (see Table 2). This highlights the problems encountered when using an unbalanced dataset. The classifier cannot distinguish positive and negative examples because the dataset contains so many more negative examples than positive examples and also because many of the positive examples are analogs of the negatives.

**Table 2:** Comparison of the PupPred with existing methods.

Method	Threshold	Ac(%)	Sn(%)	Sp(%)	MCC
GPS-PUP	High	85.44	33.07	90.18	0.1991
	Medium	82.51	44.88	85.91	0.2279
	Low	78.85	63.78	80.21	0.2864
iPUP		85.24	70.87	86.35	0.3860
		81.92	78.74	82.16	0.3777
		78.25	85.04	77.73	0.3654
PupPred^1^		85.07	30.71	89.26	0.1574
		83.15	42.52	86.29	0.2037
		77.58	54.33	79.37	0.2063
PupPred		92.97	97.27	92.66	0.6629
		88.29	98.18	87.57	0.5582
		79.90	100.00	78.44	0.4449

The thresholds of High, Medium and Low for GPS-PUP represent that the score are greater than 2.738, 2.452 and 2.111, respectively; the thresholds of High, Medium and Low for iPUP represent that the score are greater than 0.1167, 0.1044 and 0.0963, respectively; the thresholds of High, Medium and Low for PupPred represent that the SVM probability are greater than 0.8, 0.7 and 0.5.

In conclusion to this, PupPred was constructed by a balanced training set that showed better performance than GPS-PUP. It becomes obvious that the problem of prediction ability lies not only in specificity but also in sensitivity. Meanwhile, our method confirms the importance of a balanced training set for building model.

While we were working on this project, another predictor of pupylation sites (iPUP, http://cwtung.kmu.edu.tw/ipup/) was developing by the author of PupDB [16]. It was trained just like us on the same pupylation dataset extracted from PupDB database. iPUP was constructed based on the feature of the composition of *k*-spaced amino acid pairs, also used in our work. The developed predictor achieved proper performance on our testing data, but that was worse than our predictor, as can be seen from Table 2.

### Ubiquitylation Sites versus Pupylation Sites

As described in the Introduction section, ubiquitylation and pupylation are functional analogues. Several works had been done for the comparison issue between ubiquitylation and pupylation from proteasome pathways level [2], [18], [29]. In the eukaryotic proteasome pathway, ubiquitin is coupled to substrates via the carboxy group of its C-terminal glycine in a multistep reaction involving several enzymes. In contrast, the prokaryotic proteasome pathway is that the C-terminal glutamine of Pup is first deamidated to glutamine by deamidase of Pup (Dop) [14], after which Pup is coupled by PafA to the ε-amino group of a substrate lysine via an isopeptide bond [30]. Although the end-point for both the ubiquitin degradation system in eukaryotes and the Pup degradation system in prokaryotes is the proteasome, the two functionally analogous tagging systems do not share similar methods of activation and conjugation to target proteins [2], [18], [29]. We recently introduced computational algorithm to predict eukaryotic ubiquitylation sites [17], in which some ubiquitylated determinants were analyzed for performance improvement. In our subsequent work, the computational algorithms were also evaluated on the prokaryotic pupylation data that an unacceptable prediction results were obtained (see Table S2). So it will be necessary to further discuss the similarities and differences between the ubiquitin and Pup proteasome systems in terms of modified substrate.

First, to determine whether the modified substrates in eukaryotes and prokaryotes have consistent or distinct sequence properties, we calculated the relative amino acid compositions between modified sites and the non-modified sites of eukaryotes and prokaryotes, respectively. This analysis shows 7 compositional differences between eukaryotes and prokaryotes (Figure S2, small boxes). An interesting feature of modified sites is the abundance of uncharged and polar amino acids (Gln, Gly, Asn, Thr and Tyr) for eukaryotes, but for prokaryotes that is absent. In contrast, the non-polar amino acids (Val and Phe) are abundant in prokaryotes and are scarce in eukaryotes. This suggests that environment surrounding modified sites is different between prokaryotes and eukaryotes.

**Figure 5:**
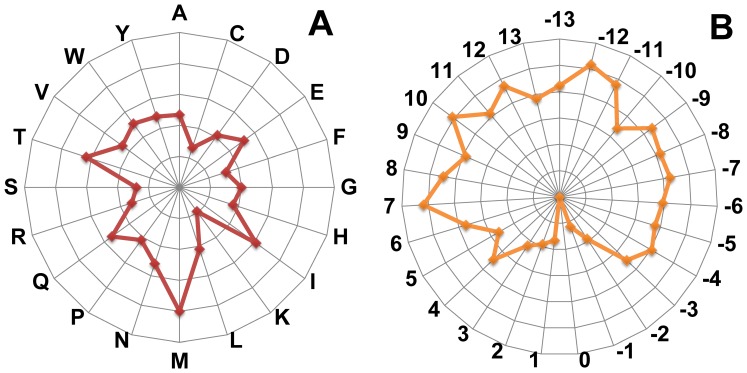
Relative importance of the different positions and amino acid residues. The importance of each residues or positions is represented by a radial vector whose length is the log2 ratio of information gain score between surrounding pupylation and ubiquitylation sites. A, the radar chart represents the log_2_ ratio of information gain score for amino acid residues between surrounding pupylation and ubiquitylation sites. B, the radar chart represents the log_2_ ratio of information gain score for positions between surrounding pupylation and ubiquitylation sites.

Second, conservation analysis is used for measuring the functionally important residues and positions between eukaryotes and prokaryotes in sequence fragments. All residues and positions in a protein are not equally important. Some are essential for the proper structure and function of a protein. Here, we used information gain approach for estimating the importance of different positions and different amino acid residues in the window based on Kullback–Leibler divergence [31]. The calculation process is described in the Procedures S1. The significance chart in Figure 5 illustrates the relative importance of the different positions and amino acids from eukaryotes and prokaryotes. As can be seen from Figure 5, the relative importance of positions and amino acid residues, both surrounding pupylation and ubiquitylation sites, are remarkably different in the sequence fragments. For instance, the residues Glu, Gln, Ile, Met, Thr and Trp are more significantly important in prokaryotes surrounding the pupylation sites than in eukaryotes surrounding the ubiquitylation sites; the positions of −12, −11, 7, 10 and 12 are more conservative in prokaryotes surrounding the pupylation sites than in eukaryotes surrounding the ubiquitylation sites.

**Figure 6:**
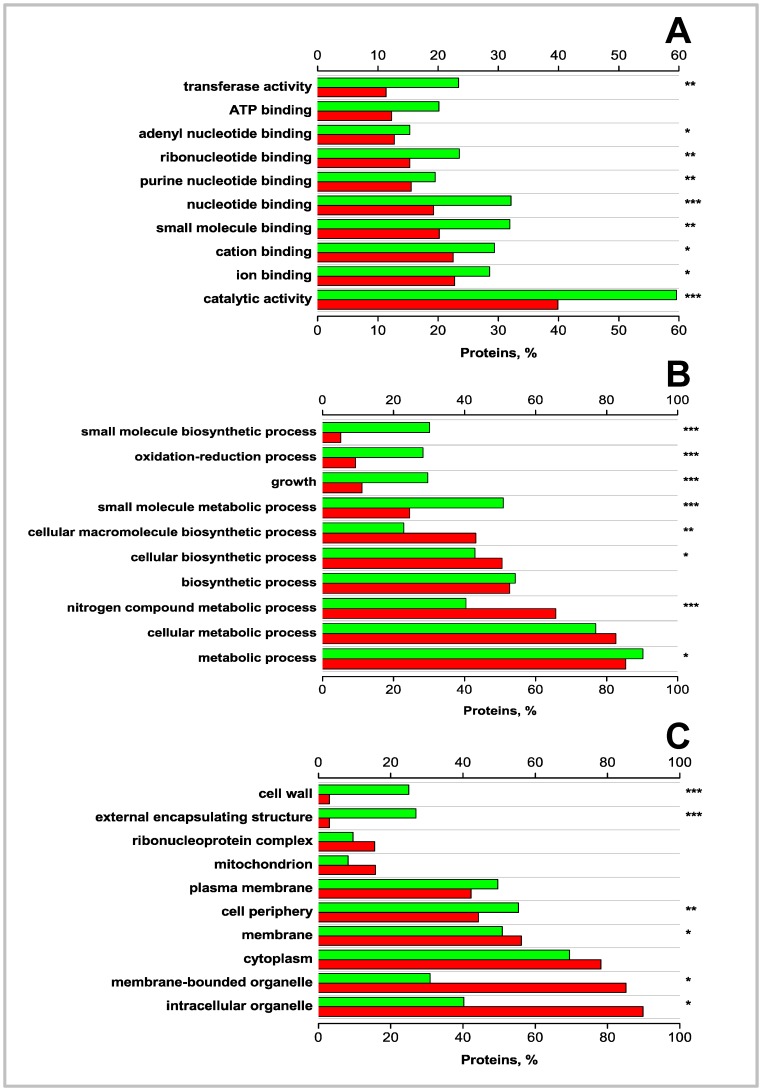
GO annotations for the highly pupylated proteins from prokaryotes proteome (red bars) with occurrence of >5% (Bonferroni corrected) as compared to the highly ubiquitylated proteins from eukaryotes proteome (green bars). Top 10 (whenever available) GO Slim terms are shown. (A) Molecular function; (B) Biological process; (C) Cellular component. The proteins are arranged in order of the decreasing fraction of proteins with a specific GO annotation present in the predicted highly pupylated dataset. *P*-values were calculated using the Fisher’s exact test (two tails) and corrected for multiple testing. ***P<0.0001; **P<0.001; *P<0.05.

Third, besides the evaluation at the sequence level, we investigated whether there are differences in the extent of functions for modified substrates between prokaryotes and eukaryotes, we took 11547 of ubiquitylated substrates in eukaryotes from PhosphoSite database [32] ((Apr 22, 2013)) and UniProtKB/Swiss-Prot database [33] ((Apr 22, 2013)) and 182 of pupylated substrates in prokaryotes from training set, and statistically analyzed the differences of biological processes, molecular functions and cellular components using Blast2GO tool [34]. The analysis of the “molecular function” annotation shows that both ubiquitylated and pupylated proteins span several functional categories (Figure 6A). These categories may be combined into two broader classes: (1) proteins involved in binding (small molecule, nucleotide, ion, ATP, etc.); (2) proteins involved in catalysis (catalytic and transferase). Among these classes, we observed significant enrichment of pupylated proteins annotated as catalytic and oxidoreductase activity, small molecule and nucleotide binding; in contrast, the ubiquitylated proteins are found that significant lack of these classes (*P<*0.0001). The “biological process” annotation shows that pupylated proteins compared with ubiquitylated proteins are enriched within such GO processes (*P*<0.0001) as metabolic, oxidation-reduction and small molecular biosynthetic (Figure 6B). Indeed, previous studies have implicated pupylated proteins in metabolic processes [13], [14]. Moreover, in contrast with the three-step biochemical reaction of eukaryotic ubiquitylation with E1, E2 and E3 ligases [35], the prokaryotic pupylation is much simpler, having only two steps [29]. The results shed light on key differences of biological process between the two pathways because of the differences of enzymology. Within the “cellular component” category, ubiquitylation proteins are prevalent within GO annotations such as cell wall and external encapsulating structure (*P*<0.0001), and cell periphery (*P*<0.05) (Figure 6C). In this regard, the results show that molecular function and biological process are more varied than cellular component.

All in all, despite using the proteasome as the end-point for proteolysis, Pup differs from ubiquitin both environmentally and functionally. These differences of modified substrate also explain the fact that substrates are targeted to prokaryotic or eukaryotic proteasomes by a fundamentally different mechanism. This systematic analysis provides a better understanding of the functional complexity and diversity of the pupylation regulation in prokaryotic.

## Discussion

Although much knowledge about pupylation has been accumulated to date, there are still numerous unanswered questions regarding specific aspects of this highly complex system. So far, no consensus sequence that determines which specific lysine of the substrate would become pupylation has been identified when non-homologous proteins are considered. In addition, the broad range of specificities of the PafA, together with the relative rigidity of their structures, raises a question about the mechanisms of substrate selection. It is difficult to assume that all substrates carry a similar preexisting structure before they bind to the components of the pupylation machinery.

Systematic analysis of the pupylated substrates along with information on the exact sites is fundamental for identifying the substrates, and can provide helpful insights into protein degradation. Here, we examine not only the sequence-derived hallmarks but also structural and evolutionary hallmarks around pupylation sites. First, our analysis of sequence preferences shows that the upstream and adjacent amino acid residues in sequence may be close to pupylation lysines in three-dimensional structure. Second, pupylation protein sequences have high propensity for exposure and flexibility. Third, there are high preferences of conservative in pupylation sequences. Moreover, we systematically compare the differences of pupylation and ubiquitylation from the environmental, conservative hallmarks and statistical analyzing of their respective gene ontology (GO) terms. The analysis suggests that pupylation site differs from ubiquitylation site both environmentally and functional characterization of substrates. These analyses provide a global landscape of the complexity and diversity of pupylation regulation in prokaryotes.

Another important result of this work is development of the pupylation sites predictor. PupPred achieved a balanced accuracy of ∼75%, and area under the ROC curve was estimated to be 83.26%. Here, we also found that unbalanced datasets present a number of different problems for machine learning methods. When only a comparatively small number of examples are available for one class, the machine learning algorithm will not have sufficient information to learn a function to distinguish the classes. Reporting of classification accuracy is also impacted by unbalanced datasets [28]. Although PupPred provides a useful alternative strategy for annotating pupylation events in prokaryotic proteomes, it has some limitations, most of which are common for almost all current prediction tools of post-translational modification site. First of all, although computational predictions indicate the possibilities that query sites can/cannot be pupylated, our predicted results have not been correlated to different cell states or tissue conditions. Second, the pupylation sites used in the training data were mostly identified by mass spectrometry methods, which may have inherent bias in terms of representing the global pupylation events and hence affect the prediction performance. As techniques like electron transfer dissociation and alternative proteases are helping to resolve technology limitations, more complete pupylation data sets will be released. We will adapt our program and prediction models as the new data become available. Third, a limitation of the data is that we have only labeled positive data, but we do not have labeled negative data (i.e. we do not know whether the non-pupylation sites are truly negatives), and therefore, if some of them are predicted as pupylation sites, we do not know whether they are false positives. For future work, we will explore other methods, such as semisupervised learning, to address these limitations.

To summarize, the development of PupPred represents an attempt to identify candidate pupylation sites based on the local sequence information. Although the number of experimentally determined pupylation sites will be growing in the future and these sites will be added to our training set to improve predictor performance, the current accuracy of PupPred is useful for predicting novel pupylation substrates as well as new sites in already known substrates. With an established link between the Pup-proteasome system and survival and persistence of Mtb [30], such predictions, especially when confirmed by experiments, would help to target the degradation of individual proteins more precisely, and may ultimately lead to development of better drugs.

## Materials and Methods

### Data Collection and Preprocessing

To construct PupPred, 182 pupylated substrates, which were previously compiled by Chun-Wei Tung [16], were downloaded from PupDB database (Mar 16 2012, http://cwtung.kmu.edu.tw/pupdb/). These 182 proteins contained 215 experimentally validated pupylation sites, which are regarded as positive samples (see Table S1). We used the same type of residue (lysine), excluding known pupylation sites as the negative samples (i.e. non-pupylation sites). Although not all these sites are necessarily true negatives, it is reasonable to believe that a large majority of them are [36]. Simultaneously, to prevent overestimation of the predictive performance, redundant sequences are removed from the training data by using a window size of 2*n*+1 for pupylation sites. We took the threshold of 30% sequence identity to filter the initial dataset. Briefly, the filtering ensured that any fragment pair in all the remaining positive and negative samples shared a sequence identity less than 30%. The data in the non-redundant training data include 190 pupylation sites and 1629 non-pupylation sites, as shown in Table 3. To perform the cross-validation, all of the non-redundant positive samples were selected to be in the positive training set. The balanced negative training set was randomly extracted from the non-redundant negative samples. However, the negative training set, which was randomly selected, might be not sufficiently response to the characteristics of the overall non-redundant negative samples. Therefore, ten negative training sets balanced with the positive sets were obtained by random extraction from the non-redundant negative samples. Meanwhile, to further evaluate the performance of PupPred and compare it with existing methods, a testing set is adopted from Xue *et al.* [15], which includes 127 pupylation sites and 1648 non-pupylation sites from 109 pupylation proteins.

**Table 3:** The statistics of training data and testing data for pupylation and non-pupylation sites.

	Training data*	Testing data
Number of proteins	182	109
Number of pupylated lysines	215 (190)	127
Number of non-pupylated lysines	2504 (1629)	1648

* Numbers in parentheses represent unique sequence after removing the redundant sequence.

### Feature Extraction and Coding

Based on sequence, structural and evolutionary information surrounding pupylation sites and non-pupylation sites, this study assesses eight types of features including binary encoding, amino acid compositions, amino acid pair compositions, grouping amino acid compositions, physicochemical properties, KNN feature, secondary structure and PSSM profile. A brief summary of the relevant features coding are:

- *Binary encoding* is generated by a 20-dimensional binary vector for each residue in the window.
- *Amino acid compositions* are generated from the frequency of 20 types of amino acids in the window, the sum of which is 1.
- *Amino acid pair compositions* are represented by the composition of *k*-spaced residue pairs [37] in the window.
- *Grouping amino acid compositions* are generated by clustering the amino acid into five groups according to amino acid properties.
- *Physicochemical properties* are encoded numerical values of properties for the residues in the window. This involves finding the significant physicochemical properties from AAIndex database [38].
- *KNN feature* [39] is generated by extracting features from its similar sequences in both positive and negative sets with a KNN algorithm.
- *Secondary structure* features for helix/strand/coil on both sides of the predicted residue, which annotates whether a helix or a strand segment (or neither) is predicted to the left or right of the residue in the center of the window.
- *PSSM profile* is generated by using PSI-BLAST [24] against the whole Swiss-Prot non-redundant database with default parameters.

Details concerning the calculation of the features are given in the Procedures S2. We note that these feature coding methods were never before used to predict pupylation sites in prokaryotic proteins.

### Feature Selection

Some of these features may not be relevant to the prediction of pupylation sites and they could be also redundant with each other. Therefore, we performed a feature selection method known as F-select [25] to remove the irrelevant and redundant features. The selection method was performed using the 10-fold cross validation for each of the ten training sets as follows. First, the F-score was calculated for each of the ten training sets. The averaged, over the ten training sets, F-score values were used to rank the features. We used a wrapper-based feature selection with the forward best first search. More specifically, for a given list of feature F =  [*f_i_* where *i* = 1, 2, …, n] sorted in the descending order by their average F-score and an empty list S that stores the selected features, we add the top-ranked feature from F to S and run SVM using feature set S in the cross validation regime. If the addition of the top ranked feature improves the average accuracy value over the ten test folds, then this feature is retained in S; otherwise it is removed. We repeat that until F is empty. Finally, the SVM classifier is trained to distinguish pupylation and non-pupylation sites on the selected feature set. The F-score of *i*th feature is defined as,
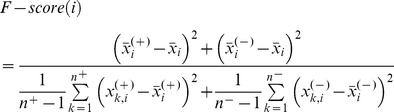
(1)where 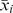, 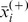 and 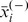 are the average value of the *i*th feature in whole, positive and negative data sets, respectively. 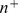 denotes the number of positive data, 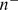 denotes the number of negative data, 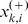 denotes the *i*th feature of the *k*th positive instance, and 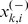 denotes the *i*th feature of the *k*th negative instance.

### SVM Learning

As a machine-learning method of binary classification, SVM aims to find a rule that best maps each member of a training set to the correct classification, which has been used for diverse prediction/classification tasks related to protein bioinformatics. Using the optimal feature coding as input, the SVM was trained to distinguish pupylation and non-pupylation sites in this study. The implemented SVM algorithm was LIBSVM (http://www.csie.ntu.edu.tw/~cjlin/libsvm) [40] and the applied kernel function was the radial basis function (RBF). In order to maximize the performance of the SVM algorithm, the penalty parameter *C* and kernel parameter were tuned based on the training set using the grid search strategy in LIBSVM.

### Evaluation Criteria and Test Procedure

We use 10-fold cross validation to assess predictions on 10 training sets. The sequences in training set are randomly divided into 10-folds, of which nine are used for training and the one for testing; each of the 10-folds is used once as the test fold. In addition, a testing set is used to assess the prediction models that are built utilizing the training set with highest cross-validated accuracy value. The binary predictions are assessed using four measures: Sensitivity (*Sn*), Specificity (*Sp*), Accuracy (*Ac*) and Matthew correlation coefficient (*MCC*).
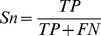
(2)

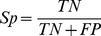
(3)

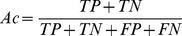
(4)

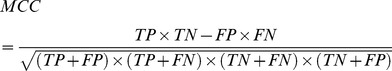
(5)where *TP* (true positives) and *TN* (true negatives) are the counts of correctly predicted pupylation and non-pupylation sites, respectively, *FP* (false positives) are non-pupylation sites that were predicted as pupylation, and *FN* (false negatives) are pupylation residues that were predicted as non-pupylation. The sensitivity, specificity and accuracy evaluate quality of predictions for the predicted native pupylation sites, native non-pupylation sites and both native pupylation and non-pupylation sites, respectively. The *MCC* evaluates the overall predictive quality. *MCC* values are between −1 and 1 with higher values for better predictions. Next, the true positive rate (i.e. *Sn*) and the false positive rate (i.e. 1−*Sp*) are calculated to draw the receiver operating characteristic (ROC) curve and we use the area under the curve (AUC) to quantify the predictive quality. Unlike the measures that assess the binary predictions, which depend on the cutoff threshold to define pupylation/non-pupylation sites, the AUC value considers all possible thresholds and thus it provides a more comprehensive evaluation.

## Supporting Information

Figure S1 **The Two Sequence Logo of the compositional biases around pupylation sites compared to the non-pupylation sites in prokaryotes.** Only amino acid residues significantly enriched and depleted (*P*<0.01; *t*-test) around pupylation sites are shown.
(TIF)

Figure S2 **Relative amino acid composition of the prokaryotes and eukaryotes.** The small box represents that they have differences of enrichment.
(TIF)

Table S1 **List of training data with associated annotation.**
(XLS)

Table S2 **The prediction performance of prokaryotic pupylated proteins from the PupDB database on the UbiProber.** UbiProber is a eukaryotic ubiquitylation prediction tool that contains four training models including *Homo sapiens*, *Mus musculus*, *Saccharomayces cerevisiae*, and *Combined*.
(DOC)

Table S3 **Secondary structure (SS) and average position-specific scoring matrix value (APV) of positions around pupylation sites and non-pupylation sites are compared via** ***P*** **-values on the paired Welch’s t-test.** There is statistical difference when *P*≤0.05, or else there isn’t significantly different.
(DOC)

Table S4 **Structural analysis of known pupylated sites.** The red row represent that the exact structural information of pupylated protein is known in the PDB.
(DOC)

Table S5 **The predictive performance of model trained with different features is compared via** ***P*** **-values of Matthew correlation coefficient on the paired Welch’s t-test.** For the entry at row *i*, column *j* of the table, there is statistical difference when P≤0.05, or else there isn’t significantly different. BE: Binary encoding; AAC: AA composition; AAPC: AA pair composition; GAAC: Grouping AA composition; PP: Physicochemical properties; KNN: *k* nearest neighbor; SS: Secondary structure; PSSM: Position specific scoring matrix. *The lower right corner of the number indicates the number of selected best feature sets by the F-score method.
(DOC)

Table S6 **Amino acid pair features of top 121 selected by feature selection of F-score method.**
(DOC)

Table S7 **Prediction performance of PupPred in 10 training sets.** *The numbers represent the average value ± standard deviation.
(DOC)

Procedures S1 **The calculation process of information gain values on different positions and different amino acid residues.**
(DOC)

Procedures S2 **Detailed feature-based sequence representation.**
(DOC)
