# Supplementary figures and images for: Systematic Analysis and Prediction of Pupylation Sites in Prokaryotic Proteins
Source: PLoS One. 2013 Sep 3;8(9):e74002. doi: 10.1371/journal.pone.0074002 (PMC3760804; doi:10.1371/journal.pone.0074002)

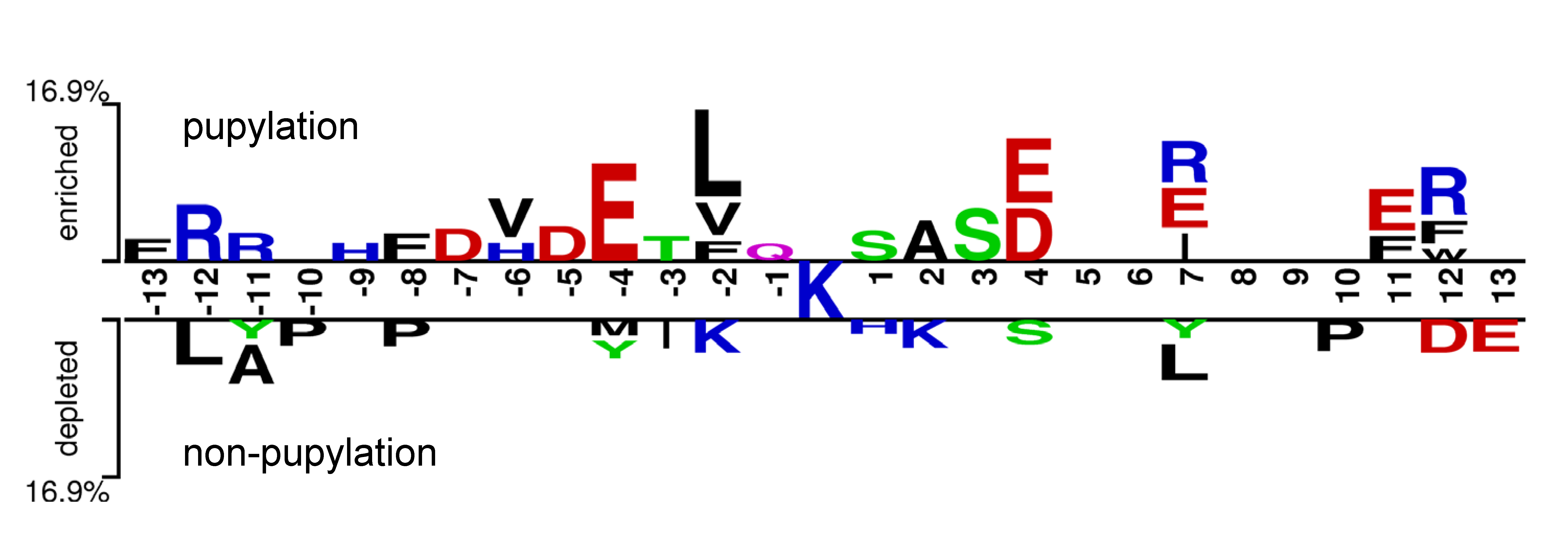

Supplement: Figure S1 — The Two Sequence Logo of the compositional biases around pupylation sites compared to the non-pupylation sites in prokaryotes. Only amino acid residues significantly enriched and depleted (P<0.01; t-test) around pupylation sites are shown. (TIF) [file pone.0074002.s001.tif]

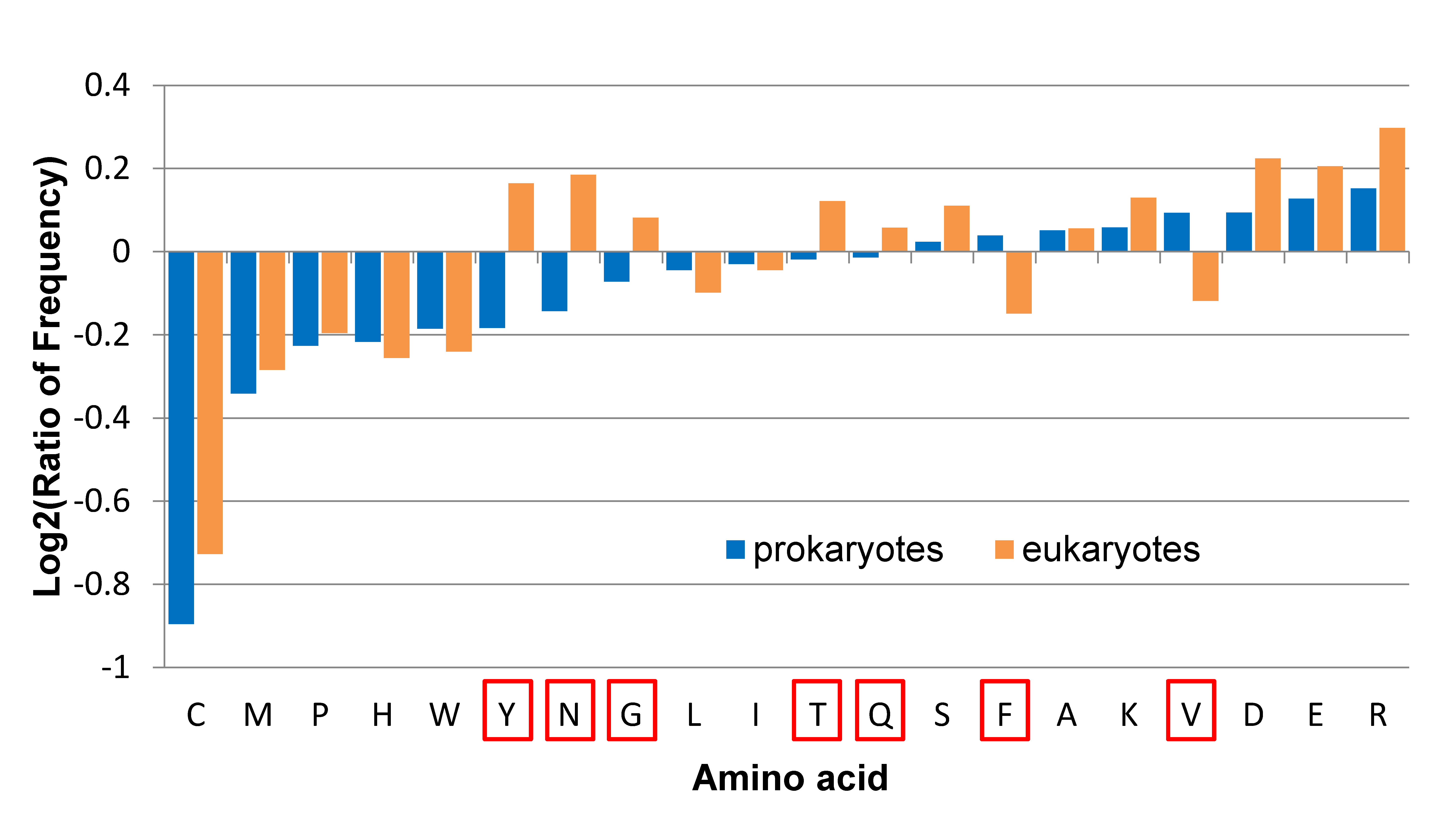

Supplement: Figure S2 — Relative amino acid composition of the prokaryotes and eukaryotes. The small box represents that they have differences of enrichment. (TIF) [file pone.0074002.s002.tif]
