# Supplementary material for: Systematic Analysis and Prediction of Pupylation Sites in Prokaryotic Proteins
Source: PLoS One. 2013 Sep 3;8(9):e74002. doi: 10.1371/journal.pone.0074002 (PMC3760804; doi:10.1371/journal.pone.0074002)
Supplement: Table S2 — The prediction performance of prokaryotic pupylated proteins from the PupDB database on the UbiProber. UbiProber is a eukaryotic ubiquitylation prediction tool that contains four training models including Homo sapiens, Mus musculus, Saccharomayces cerevisiae, and Combined. (DOC) [file pone.0074002.s004.doc]

**Table S2. The prediction performance of prokaryotic pupylated proteins from the PupDB database on the UbiProber. UbiProber is a eukaryotic ubiquitylation prediction tool that contains four training models including *Homo sapiens*, *Mus musculus*, *Saccharomayces cerevisiae*, and *Combined*.**

| Training model | *Sn*(%) | *Sp*(%) | *Ac*(%) | *MCC* |
| --- | --- | --- | --- | --- |
| *H. sapiens* | 53.65 | 61.26 | 57.46 | 0.1495 |
| *M. musculus* | 60.10 | 65.20 | 62.66 | 0.2536 |
| *S. cerevisiae* | 63.12 | 57.83 | 60.48 | 0.2099 |
| *Combined* | 58.50 | 70.66 | 64.58 | 0.2940 |
