# Supplementary material for: Systematic Analysis and Prediction of Pupylation Sites in Prokaryotic Proteins
Source: PLoS One. 2013 Sep 3;8(9):e74002. doi: 10.1371/journal.pone.0074002 (PMC3760804; doi:10.1371/journal.pone.0074002)
Supplement: Table S3 — Secondary structure (SS) and average position-specific scoring matrix value (APV) of positions around pupylation sites and non-pupylation sites are compared via P -values on the paired Welch’s t-test. There is statistical difference when P≤0.05, or else there isn’t significantly different. (DOC) [file pone.0074002.s005.doc]

**Table S3. Secondary structure (SS) and average position-specific scoring matrix value (APV) of positions around pupylation sites and non-pupylation sites are compared via *P*-values on the paired Welch’s t-test. There is statistical difference when *P*$\leq$0.05, or else there isn’t significantly different.**

|  | **-13** | **-12** | **-11** | **-10** | **-9** | **-8** | **-7** | **-6** | **-5** | **-4** | **-3** | **-2** | **-1** | **0** |
| --- | --- | --- | --- | --- | --- | --- | --- | --- | --- | --- | --- | --- | --- | --- |
| ***(a)* *P*-value of comparison of pupylation with non-pupylation at upstream positions** | | | | | | | | | | | | | | |
| **SS** | **0.05212** | **0.03647** | **0.08361** | **0.08862** | **1.924e-03** | **0.07854** | **0.1033** | **3.28e-03** | **0.0259** | **0.06321** | **0.05648** | **7.258e-06** | **5.341e-06** | **8.127e-03** |
| **APV** | **2.956e-04** | **1.281e-04** | **3.984e-03** | **5.216e-07** | **3.058e-04** | **0.01442** | **0.05288** | **6.024e-04** | **0.01169** | **4.028e-04** | **1.899e-04** | **5.324e-06** | **0.08231** | **0.09518** |
| ***(b)* *P*-value of comparison of pupylation with non-pupylation at downstream positions** | | | | | | | | | | | | | | |
|  | **13** | **12** | **11** | **10** | **9** | **8** | **7** | **6** | **5** | **4** | **3** | **2** | **1** |  |
| **SS** | **0.1058** | **0.1123** | **0.2087** | **0.05984** | **0.04893** | **0.07419** | **0.09874** | **6.283e-04** | **0.1004** | **0.02549** | **0.1473** | **0.01946** | **0.02017** |  |
| **APV** | **3.694e-06** | **0.03659** | **0.01764** | **7.822e-04** | **3.562e-03** | **8.416e-07** | **2.684e-04** | **0.02255** | **0.07215** | **3.393e-04** | **6.342e-06** | **8.234e-04** | **5.554e-06** |  |
