# Supplementary material for: Systematic Analysis and Prediction of Pupylation Sites in Prokaryotic Proteins
Source: PLoS One. 2013 Sep 3;8(9):e74002. doi: 10.1371/journal.pone.0074002 (PMC3760804; doi:10.1371/journal.pone.0074002)
Supplement: Table S4 — Structural analysis of known pupylated sites. The red row represent that the exact structural information of pupylated protein is known in the PDB. (DOC) [file pone.0074002.s006.doc]

**Table S4.** **Structural analysis of known pupylated sites.** The red row represent that the exact structural information of pupylated protein is known in the PDB.

| UniProt ID | PDB chain | Residues From protein sequence | Residues from PDB chain | BLAST identity (%) | Pupylated site(s) | Site on aligned PDB chain (e-extended strand, c-coil, h-helix) | Additional information (if available) |
| --- | --- | --- | --- | --- | --- | --- | --- |
| A0QNF6 | 1w74_A | 3-175 | 19-191 | 89 | K147 | V164 | Residue is not K in the PDB chain |
| A0QQU5 | 3rtk_A | 1-527 | 1-527 | 94 | K116,K132 | K116(h),K132(h) | -- |
| A0QS98 | 3agp_A | 1-395 | 285-677 | 75 | K188 | A471 | Residue is not K in the PDB chain |
| A0QSH8 | 2cdn_A | 1-181 | 21-201 | 78 | K23 | K43(h) | -- |
| A0QUY3 | 1w0d_A | 6-340 | 3-337 | 82 | K313 | R310 | Residue is not K in the PDB chain |
| A0QUZ0 | 3h5h_A | 2-186 | 5-189 | 87 | K61 | R64 | Residue is not K in the PDB chain |
| A0QWT3 | 3tde_A | 3-399 | 9-408 | 87 | K341,K396 | K350(h),K405(h) | -- |
| A0QZ54 | 3m9b_A | 1-238 | 1-234 | 82 | K595 | N/A | Site is not covered by structure |
| A0R066 | 3jz6_A | 2-368 | 7-373 | 100 | K299 | K304(h) | -- |
| A0R079 | 2bvc_A | 3-478 | 11-486 | 84 | K14 | K22(h) | -- |
| A0R0B2 | 2qj3_A | 1-290 | 36-322 | 74 | K157 | K193(h) | -- |
| A0R0B3 | 1klp_A | 1-95 | 1-95 | 92 | K58,K79 | K58(h),K79(h) | -- |
| A0R0B4 | 2wgd_A | 1-416 | 1-416 | 86 | K53 | K53(h) | -- |
| A0R0B5 | 2gp6_A | 4-417 | 21-434 | 79 | K84 | R101 | Residue is not K in the PDB chain |
| A0R0W1 | 1zj8_A | 18-569 | 15-566 | 83 | K458 | R454 | Residue is not K in the PDB chain |
| A0R1V9 | 2bmx_A | 1-194 | 1-194 | 80 | K25,K29,  K41 | K25(c),K29(e),D41 | 41K>D in the struture |
| A0R220 | 2d1f_A | 1-340 | 1-340 | 86 | K151 | K151(h) | -- |
| A0R2G5 | 3fsx_A | 1-314 | 6-319 | 74 | K299 | A304 | Residue is not K in the PDB chain |
| A0R4C9 | 3hwi_A | 1-277 | 22-298 | 88 | K67 | K88(h) | -- |
| A0R518 | 3uve_A | 1-279 | 5-286 | 78 | K65 | K72(h) | -- |
| A0R566 | 1ym3_A | 2-205 | 10-213 | 72 | K11 | K19(h) | -- |
| A0R647 | 3oj5_A | 1-181 | 1-181 | 71 | K10 | K10(h) | -- |
| A0R652 | 1gn2_A | 1-207 | 1-207 | 80 | K38,K90 | K38(h),K90(e) | -- |
| A0R7G6 | 1gr0_A | 1-363 | 1-367 | 85 | K65 | K73(c) | -- |
| A4ZHR8 | 2ziz_A | 4-485 | 11-495 | 87 | K464 | K474(h) | -- |
| O53442 | 1za0_A | 1-275 | 1-275 | 100 | K145 | K145(e) | -- |
| O53665 | 3lls_A | 1-454 | 22-475 | 100 | K168,K381 | K189(e),K402(c) | -- |
| O53871 | 3svk_A | 1-403 | 5-407 | 90 | K189 | K193(c) | -- |
| P02358 | 1p6g_F | 1-135 | 1-135 | 100 | K35 | K35(e) | -- |
| P02359 | 1vs5_G | 1-179 | 1-179 | 100 | K56,K131 | K56(e),K131(e) | -- |
| P02413 | 1p85_J | 1-144 | 1-144 | 100 | K141 | K141(e) | -- |
| P04079 | 1gpm_A | 1-525 | 1-525 | 100 | K437 | K437(h) | -- |
| P06968 | 1dud_A | 1-151 | 2-152 | 100 | K15 | K16(c) | -- |
| P07012 | 1gqe_A | 1-365 | 1-365 | 99 | K162 | K162(e) | -- |
| P08200 | 3dms_A | 5-416 | 14-426 | 75 | K4,K273 | N283 | Residue is not K in the PDB chain |
| P08839 | 2hwg_A | 1-575 | 1-575 | 100 | K174 | K174(c) | -- |
| P09030 | 1ako_A | 1-268 | 1-268 | 100 | K141 | K141(e) | -- |
| P09372 | 1dkg_A | 1-197 | 1-197 | 99 | K43,K66 | K43(h),K66(h) | -- |
| P09621 | 1hx5_A | 2-100 | 1-99 | 100 | K100 | K99(e) | -- |
| P0A520 | 3rtk_A | 1-540 | 1-540 | 100 | K132 | K132(h) | -- |
| P0A5H3 | 1f61_A | 2-428 | 3-429 | 100 | K334 | K335(h) | -- |
| P0A5U4 | 1g18_A | 657-790 | 219-350 | 100 | K762 | K322(h) | -- |
| P0A6F5 | 1grl_A | 1-548 | 1-548 | 99 | K65,K168,K321 | K65(h),K168(h),K321(e) | -- |
| P0A6Y8 | 2kho_A | 1-605 | 1-605 | 99 | K635,K637 | N/A | Site is not covered by structure |
| P0A715 | 1o60_A | 1-283 | 1-283 | 81 | K79 | K79(h) | -- |
| P0A715 | 2qkf_A | 6-275 | 3-272 | 71 | K79 | K76(h) | -- |
| P0A7V8 | 1vs5_D | 1-206 | 1-206 | 100 | K156,K167 | K156(h),K167(e) | -- |
| P0A853 | 2c44_A | 1-471 | 1-471 | 99 | K156 | K156(c) | -- |
| P0A8A0 | 1kon_A | 1-246 | 4-249 | 100 | K192 | K195(e) | -- |
| P0A9B2 | 1s7c_A | 1-331 | 1-331 | 100 | K249 | K249(e) | -- |
| P0A9D8 | 3tk8_A | 2-271 | 44-314 | 76 | K100 | A143 | Residue is not K in the PDB chain |
| P0A9Y6 | 3i2z_B | 1-69 | 3-71 | 84 | K9 | K11(e) | -- |
| P0A9Y6 | 2l15_A | 3-69 | 4-70 | 70 | K9 | K10(e) | -- |
| P0ABT2 | 4dyu_A | 1-167 | 4-170 | 79 | K105 | K108(e) | -- |
| P0AGD3 | 1isa_A | 2-193 | 1-192 | 100 | K44 | K43(c) | -- |
| P0CG99 | 1pem_A | 11-722 | 3-714 | 71 | K310 | E302 | Residue is not K in the PDB chain |
| P11447 | 1tj7_A | 1-457 | 1-457 | 100 | K95 | K95(h) | -- |
| P17670 | 1gn2_A | 1-207 | 1-207 | 99 | K202 | K202(e) | -- |
| P23869 | 1lop_A | 1-164 | 1-164 | 99 | K60 | K60(e) | -- |
| P27302 | 2r5n_A | 1-663 | 1-663 | 100 | K316,K347 | K316(h),K347(h) | -- |
| P28861 | 2xnj_A | 1-247 | 11-266 | 94 | K135 | K154(e) | -- |
| P36683 | 1l5j_A | 1-865 | 1-865 | 100 | K77 | K77(e) | -- |
| P37760 | 1kbz_A | 1-299 | 1-299 | 84 | K245 | E245 | Residue is not K in the PDB chain |
| P38489 | 3hzn_A | 1-217 | 4-220 | 88 | K21,K62 | K24(e),K65(e) | -- |
| P40874 | 2uzz_A | 1-372 | 1-372 | 99 | K357 | K357(e) | -- |
| P52061 | 1k7k_A | 1-197 | 23-219 | 100 | K3 | N/A | Site is not covered by structure |
| P60176 | 2ziz_A | 1-495 | 1-495 | 100 | K474 | K474(h) | -- |
| P63345 | 3m9b_A | 1-234 | 1-234 | 100 | K591 | N/A | Site is not covered by structure |
| P63458 | 2qj3_A | 1-302 | 21-322 | 100 | K173 | K193(h) | -- |
| P65277 | 3h5h_A | 2-186 | 5-189 | 100 | K154 | K157(c) | -- |
| P66902 | 2d1f_A | 1-360 | 1-360 | 100 | K151 | K151(h) | -- |
| P68919 | 1b75_A | 1-94 | 1-94 | 100 | K34 | K34(e) | -- |
| P69440 | 2cdn_A | 1-181 | 21-201 | 100 | K94 | K114(h) | -- |
| P69441 | 1ake_A | 1-214 | 1-214 | 100 | K47,K50,  K136,K141 | K47(e),K50(h),K136(c),  K141(e) | -- |
| P71703 | 1gr0_A | 1-367 | 1-367 | 100 | K73 | K73(c) | -- |
| P77395 | 3qdn_A | 1-284 | 4-287 | 94 | K172,K256 | K175(c),K259(h) | -- |
| P77899 | 3tde_A | 1-403 | 6-408 | 99 | K345 | K350(h) | -- |
| P96382 | 3foq_A | 1-495 | 9-503 | 100 | K362 | K370(e) | -- |
| Q10682 | 2asf_A | 1-137 | 1-137 | 99 | K47 | K47(c) | -- |
| Q46856 | 1oj7_A | 1-387 | 22-408 | 99 | K43 | K64(h) | -- |
