# Supplementary material for: Systematic Analysis and Prediction of Pupylation Sites in Prokaryotic Proteins
Source: PLoS One. 2013 Sep 3;8(9):e74002. doi: 10.1371/journal.pone.0074002 (PMC3760804; doi:10.1371/journal.pone.0074002)
Supplement: Table S5 — The predictive performance of model trained with different features is compared via P -values of Matthew correlation coefficient on the paired Welch’s t-test. For the entry at row i, column j of the table, there is statistical difference when P≤0.05, or else there isn’t significantly different. BE: Binary encoding; AAC: AA composition; AAPC: AA pair composition; GAAC: Grouping AA composition; PP: Physicochemical properties; KNN: k nearest neighbor; SS: Secondary structure; PSSM: Position specific scoring matrix. *The lower right corner of the number indicates the number of selected best feature sets by the F-score method. (DOC) [file pone.0074002.s007.doc]

**Table S5 The predictive performance of model trained with different features is compared via *P*-values of Matthew correlation coefficient on the paired Welch’s t-test.** For the entry at row *i*, column *j* of the table, there is statistical difference when *P*≤0.05, or else there isn’t significantly different. BE: Binary encoding; AAC: AA composition; AAPC: AA pair composition; GAAC: Grouping AA composition; PP: Physicochemical properties; KNN: *k* nearest neighbor; SS: Secondary structure; PSSM: Position specific scoring matrix. * The lower right corner of the number indicates the number of selected best feature sets by the F-score method.

|  | BE | AAC | AAPC | GAAC | PP | KNN | SS | PSSM | *BE_233_ | PSSM_134_ | AAPC_121_ |
| --- | --- | --- | --- | --- | --- | --- | --- | --- | --- | --- | --- |
| BE | 1.000 | 0.7010 | 0.3760 | 0.8705 | 0.8645 | 0.8865 | 0.2306 | 0.6284 | 3.303e-06 | 0.01585 | 1.780e-08 |
| AAC |  | 1.000 | 0.08283 | 0.8615 | 0.4356 | 0.5952 | 0.08751 | 0.8331 | 5.399e-11 | 9.558e-06 | 5.266e-15 |
| AAPC |  |  | 1.000 | 0.2822 | 0.3799 | 0.1375 | 0.009121 | 0.06724 | 1.948e-06 | 0.06218 | 2.761e-09 |
| GAAC |  |  |  | 1.000 | 0.7165 | 0.9397 | 0.3093 | 0.7804 | 1.622e-06 | 0.008712 | 8.566e-09 |
| PP |  |  |  |  | 1.000 | 0.6423 | 0.06541 | 0.3654 | 9.189e-08 | 0.003941 | 1.294e-10 |
| KNN |  |  |  |  |  | 1.000 | 0.02408 | 0.4645 | 4.801e-11 | 1.545e-05 | 2.426e-15 |
| SS |  |  |  |  |  |  | 1.000 | 0.1431 | 5.576e-12 | 2.744e-07 | 6.037e-16 |
| PSSM |  |  |  |  |  |  |  | 1.000 | 6.476e-11 | 8.769e-06 | 8.887e-15 |
| BE_233_ |  |  |  |  |  |  |  |  | 1.000 | 7.091e-07 | 2.797e-05 |
| PSSM_134_ |  |  |  |  |  |  |  |  |  | 1.000 | 1.510e-11 |
| AAPC_121_ |  |  |  |  |  |  |  |  |  |  | 1.000 |
