# Supplementary material for: Systematic Analysis and Prediction of Pupylation Sites in Prokaryotic Proteins
Source: PLoS One. 2013 Sep 3;8(9):e74002. doi: 10.1371/journal.pone.0074002 (PMC3760804; doi:10.1371/journal.pone.0074002)
Supplement: Table S6 — Amino acid pair features of top 121 selected by feature selection of F-score method. (DOC) [file pone.0074002.s008.doc]

**Table S6. Amino acid pair features of top 121 selected by feature selection of F-score method**.

| **Res#** | **Features** | **F-score** | **Res#** | **Features** | **F-score** |
| --- | --- | --- | --- | --- | --- |
| 1 | RP | 0.0023 | 62 | RxxxV | 0.0033 |
| 2 | RV | 0.0031 | 63 | DxxxR | 0.0045 |
| 3 | CG | 0.0027 | 64 | QxxxW | 0.0014 |
| 4 | HD | 0.0022 | 65 | GxxxM | 0.0021 |
| 5 | IS | 0.0047 | 66 | GxxxY | 0.0034 |
| 6 | LL | 0.0039 | 67 | HxxxI | 0.0028 |
| 7 | LK | 0.0021 | 68 | LxxxA | 0.0029 |
| 8 | LP | 0.0031 | 69 | KxxxD | 0.0069 |
| 9 | KC | 0.0015 | 70 | KxxxS | 0.0036 |
| 10 | KK | 0.0000 | 71 | MxxxA | 0.0025 |
| 11 | KP | 0.0031 | 72 | MxxxK | 0.0011 |
| 12 | PG | 0.0031 | 73 | FxxxH | 0.0015 |
| 13 | SN | 0.0020 | 74 | PxxxN | 0.0026 |
| 14 | TH | 0.0022 | 75 | PxxxG | 0.0024 |
| 15 | WE | 0.0017 | 76 | YxxxK | 0.0030 |
| 16 | YA | 0.0016 | 77 | VxxxC | 0.0016 |
| 17 | VR | 0.0048 | 78 | AxxxxD | 0.0057 |
| 18 | VM | 0.0030 | 79 | AxxxxC | 0.0020 |
| 19 | DxD | 0.0041 | 80 | AxxxxE | 0.0037 |
| 20 | CxA | 0.0018 | 81 | RxxxxH | 0.0030 |
| 21 | CxL | 0.0017 | 82 | DxxxxP | 0.0025 |
| 22 | ExA | 0.0041 | 83 | CxxxxN | 0.0006 |
| 23 | GxR | 0.0025 | 84 | CxxxxE | 0.0015 |
| 24 | GxP | 0.0041 | 85 | IxxxxI | 0.0035 |
| 25 | HxN | 0.0018 | 86 | IxxxxM | 0.0018 |
| 26 | IxP | 0.0044 | 87 | LxxxxC | 0.0017 |
| 27 | LxK | 0.0075 | 88 | KxxxxT | 0.0011 |
| 28 | LxM | 0.0018 | 89 | MxxxxE | 0.0028 |
| 29 | KxK | 0.0012 | 90 | PxxxxH | 0.0019 |
| 30 | MxE | 0.0024 | 91 | SxxxxR | 0.0023 |
| 31 | FxE | 0.0025 | 92 | SxxxxS | 0.0044 |
| 32 | PxQ | 0.0018 | 93 | TxxxxW | 0.0014 |
| 33 | TxH | 0.0033 | 94 | WxxxxD | 0.0016 |
| 34 | TxM | 0.0024 | 95 | WxxxxT | 0.0016 |
| 35 | TxP | 0.0023 | 96 | YxxxxG | 0.0027 |
| 36 | WxA | 0.0022 | 97 | VxxxxD | 0.0044 |
| 37 | YxI | 0.0018 | 98 | AxxxxxR | 0.0041 |
| 38 | AxxL | 0.0022 | 99 | RxxxxxC | 0.0011 |
| 39 | AxxF | 0.0025 | 100 | NxxxxxK | 0.0011 |
| 40 | NxxQ | 0.0023 | 101 | DxxxxxA | 0.0055 |
| 41 | NxxG | 0.0039 | 102 | CxxxxxD | 0.0014 |
| 42 | DxxF | 0.0049 | 103 | CxxxxxG | 0.0011 |
| 43 | CxxA | 0.0018 | 104 | GxxxxxM | 0.0024 |
| 44 | CxxE | 0.0012 | 105 | HxxxxxH | 0.0016 |
| 45 | GxxC | 0.0014 | 106 | IxxxxxS | 0.0025 |
| 46 | HxxL | 0.0020 | 107 | MxxxxxA | 0.0028 |
| 47 | IxxA | 0.0017 | 108 | MxxxxxQ | 0.0016 |
| 48 | IxxM | 0.0024 | 109 | MxxxxxG | 0.0034 |
| 49 | LxxC | 0.0014 | 110 | FxxxxxM | 0.0014 |
| 50 | LxxP | 0.0035 | 111 | PxxxxxP | 0.0024 |
| 51 | LxxT | 0.0023 | 112 | PxxxxxS | 0.0017 |
| 52 | KxxI | 0.0014 | 113 | SxxxxxG | 0.0027 |
| 53 | MxxT | 0.0022 | 114 | TxxxxxG | 0.0036 |
| 54 | FxxD | 0.0042 | 115 | TxxxxxS | 0.0023 |
| 55 | FxxF | 0.0025 | 116 | WxxxxxA | 0.0032 |
| 56 | PxxQ | 0.0027 | 117 | WxxxxxD | 0.0016 |
| 57 | TxxG | 0.0031 | 118 | WxxxxxK | 0.0016 |
| 58 | YxxY | 0.0026 | 119 | WxxxxxT | 0.0010 |
| 59 | VxxY | 0.0030 | 120 | YxxxxxK | 0.0015 |
| 60 | AxxxL | 0.0022 | 121 | VxxxxxR | 0.0075 |
| 61 | RxxxM | 0.0032 |  |  |  |
