# Supplementary material for: Systematic Analysis and Prediction of Pupylation Sites in Prokaryotic Proteins
Source: PLoS One. 2013 Sep 3;8(9):e74002. doi: 10.1371/journal.pone.0074002 (PMC3760804; doi:10.1371/journal.pone.0074002)
Supplement: Table S7 — Prediction performance of PupPred in 10 training sets. *The numbers represent the average value ± standard deviation. (DOC) [file pone.0074002.s009.doc]

**Table S7.** **Prediction performance of PupPred in 10 training sets.** *The numbers represent the average value ± standard deviation.

| Training set | AUC(%) | Ac(%) | Sn(%) | Sp(%) | MCC |
| --- | --- | --- | --- | --- | --- |
| 1 | 81.48 | 74.21 | 74.23 | 74.19 | 0.4868 |
| 2 | 84.41 | 76.84 | 77.01 | 76.67 | 0.5397 |
| 3 | 82.68 | 75.00 | 75.19 | 74.81 | 0.5026 |
| 4 | 83.69 | 76.07 | 76.95 | 75.19 | 0.5239 |
| 5 | 84.89 | 77.24 | 78.16 | 76.32 | 0.5503 |
| 6 | 83.24 | 75.96 | 76.33 | 75.59 | 0.5238 |
| 7 | 84.04 | 76.58 | 77.05 | 76.11 | 0.5344 |
| 8 | 82.05 | 74.63 | 74.95 | 74.31 | 0.4974 |
| 9 | 81.97 | 74.67 | 75.31 | 74.03 | 0.4974 |
| 10 | 84.18 | 76.62 | 77.22 | 76.02 | 0.5344 |
| mean | 83.26±1.17* | 75.78±1.07 | 76.24±1.25 | 75.32±0.96 | 0.5191±0.0215 |
