# Supplementary material for: Systematic Analysis and Prediction of Pupylation Sites in Prokaryotic Proteins
Source: PLoS One. 2013 Sep 3;8(9):e74002. doi: 10.1371/journal.pone.0074002 (PMC3760804; doi:10.1371/journal.pone.0074002)
Supplement: Procedures S1 — The calculation process of information gain values on different positions and different amino acid residues. (DOC) [file pone.0074002.s010.doc]

**Procedures S1. The calculation process of information gain values on different positions and different amino acid residues.**

Information Gain (IG) was employed to distinguish the importance of different positions and different amino acid residues for ubiquitylation and pupylation sequence fragments. Here, we extracted IG scores of the positions and amino acid residues in the following procedure.

- IG procedure: Information gain measures the decrease in entropy when a given feature is used to group values of another (class) feature. The entropy of a feature *X* is defined as

(Eq. 1)

where {*x*_i_} (in our case the positive sample or negative sample) is a set of values of *X* and *P*(*x*_i_) is the prior probability of *x*_i_. The conditional entropy of *X*, given another feature *Y* (when extract IG scores of position it presents the amino acid type; when extract IG scores of amino acid residue it presents the amino acid frequency) is defined as

(Eq. 2)

where *P*(*x*_i_|*y*_j_) is the posterior probability of *X* given the value *y*_i_ of *Y*. The amount by which the entropy of *X* decreases reflects additional information about *X* provided by *Y* and is called information gain

$\mathrm{IG}\text{(}\mathrm{X}|\mathrm{Y}\text{)}=\mathrm{H}\text{(}\mathrm{X}\text{)}-\mathrm{H}\text{(}\mathrm{X}|\mathrm{Y}\text{)}$ (Eq. 3)

According to this measure, *Y* has stronger correlation with *X* than with *Z* if IG(*X|Y*)>IG(*Z*|*Y*).

- Calculated IG score of positions and amino acid residues: (1) The 20 amino acid residues (A, C, D, E, F, G, H, I, K, L, M, N, P, Q, R, S, T, V, W, Y) are coded into digital from 1 to 20. The query sequences segments are coded into 27 dimension digital sequence. (2) Calculate the amino acid frequency in the sequence surrounding the query site (the site itself is not counted). The query sequences are also coded into 20 dimension feature. (3) Calculate the IG score of positions by (1) and IG procedure; calculate the IG score of amino acid residues by (2) and IG procedure. We rank the corresponding positions and amino acid residues by IG scores, and select the key positions and key amino acid residues. The selection process is described later.

Using this method, we obtained the ranking list of IG scores of 27 positions and 20 amino acid residues. IG scores of positions and amino acids for ubiquitylation and pupylation sequence fragments are summarized in Table SS2. Note that the larger IG scores of feature, the better the importance of feature.

**Table SS2. A summary of IG score of positions and amino acid residues for ubiquitylation and pupylation sequence fragments.**

| Positions | Posttranslational modification | | Residues | Posttranslational modification | |
| --- | --- | --- | --- | --- | --- |
|  | ubiquitylation | pupylation |  | ubiquitylation | pupylation |
| -13 | 0.0005 | 0.0092 | A | 0.0009 | 0.0057 |
| -12 | 0.0004 | 0.0146 | C | 0.0031 | 0.0050 |
| -11 | 0.0004 | 0.0117 | D | 0.0009 | 0.0041 |
| -10 | 0.0006 | 0.0062 | E | 0.0006 | 0.0055 |
| -9 | 0.0004 | 0.0091 | F | 0.0005 | 0.0011 |
| -8 | 0.0006 | 0.0100 | G | 0.0012 | 0.0049 |
| -7 | 0.0007 | 0.0133 | H | 0.0017 | 0.0051 |
| -6 | 0.0009 | 0.0131 | I | 0.0003 | 0.0046 |
| -5 | 0.0010 | 0.0132 | K | 0.0037 | 0.0035 |
| -4 | 0.0014 | 0.0232 | L | 0.0009 | 0.0039 |
| -3 | 0.0016 | 0.0181 | M | 0.0001 | 0.0049 |
| -2 | 0.0033 | 0.0124 | N | 0.0002 | 0.0017 |
| -1 | 0.0045 | 0.0103 | P | 0.0011 | 0.0050 |
| 0 | 0 | 0.0000 | Q | 0.0001 | 0.0012 |
| 1 | 0.0028 | 0.0090 | R | 0.0025 | 0.0059 |
| 2 | 0.0029 | 0.0110 | S | 0.0010 | 0.0017 |
| 3 | 0.0016 | 0.0073 | T | 0.0002 | 0.0032 |
| 4 | 0.0013 | 0.0141 | V | 0.0006 | 0.0033 |
| 5 | 0.0010 | 0.0063 | W | 0.0001 | 0.0007 |
| 6 | 0.0006 | 0.0083 | Y | 0.0006 | 0.0040 |
| 7 | 0.0005 | 0.0173 |  |  |  |
| 8 | 0.0005 | 0.0103 |  |  |  |
| 9 | 0.0005 | 0.0072 |  |  |  |
| 10 | 0.0004 | 0.0119 |  |  |  |
| 11 | 0.0005 | 0.0080 |  |  |  |
| 12 | 0.0005 | 0.0129 |  |  |  |
| 13 | 0.0005 | 0.0067 |  |  |  |

**REFERENCES**

1. Tumminello M, Lillo F, Mantegna RN: **Kullback-Leibler distance as a measure of the information filtered from multivariate data**. *Phys Rev E* 2007, **76**(3).

2. Ke C, Yingfu J, Li D, Kurgan L: **Prediction of integral membrane protein type by collocated hydrophobic amino acid pairs**. *J Comput Chem* 2009, **30**(1).
