# Supplementary material for: Systematic Analysis and Prediction of Pupylation Sites in Prokaryotic Proteins
Source: PLoS One. 2013 Sep 3;8(9):e74002. doi: 10.1371/journal.pone.0074002 (PMC3760804; doi:10.1371/journal.pone.0074002)
Supplement: Procedures S2 — Detailed feature-based sequence representation. (DOC) [file pone.0074002.s011.doc]

**Procedures S2. Detailed feature-based sequence representation.**

For each residue in the input sequence we compute the following features using the sliding window of size 27:

- *Binary encoding* generated by a 20-dimensional binary vector for each residue in the window. The 20 different amino acids are considered in binary encoding, which are ordered as ACDEFGHIKLMNPQRSTVWY. Briefly, each amino acid is represented by a 20-dimensional binary vector, e.g. A (10000000000000000000), C (01000000000000000000), … Y (00000000000000000001), etc. (total of 20×27 = 540 features).
- *Amino acid compositions* are generated from the frequency of 20 types of amino acid in the window. We calculated the amino acid frequencies in the sequence surrounding the pupylation sites (the site itself is not counted). There are 20 types of amino acids, and thus 20 frequencies are calculated, the sum of which is 1 (total of 20×1 = 20 features).
- *Amino acid pair compositions* are represented by the composition of *k*-spaced residue pairs in the window. Taking *k* = 0 as an example, there are 400 0-spaced residue pairs (i.e., AA, AC, AD, …, YY). Then, a feature vector can be defined as

The value of each feature denotes the composition of the corresponding residue pair in the fragment. For instance, if the residue pair AA appears *m* times in the window, the composition of the residue pair AA is equal to m divided by the total number of 0-spaced residue pairs (*N*) in the fragment. For *k*=0, 1, 2, 3, 4 and 5, the value of *N* is 26, 25, 24, 23 and 22, respectively. Considering that the *k*-spaced encoding was performed over *k*= 0, 1, 2, 3, 4 and 5 in this study (total of 400×6 = 2400 features).

- *Grouping amino acid compositions* generated by clustering the amino acid into five groups according to their properties. The five-class grouping method is used to 20 amino acids into subgroups that capture their biochemistry properties. Five-class grouping methods can be based on charge, hydrophobicity , surface exposure , disorder and flexibility (total of 5×27 = 135 features). Charge groups including residues (positively charged residues: K, R, H; negatively charged residues: D, E; neutral residues: A, C, F, G, I, L, M, N, P, Q, S, T, V, W, Y); hydrophobicity groups including residues (hydrophobic residues: A, F, G, I, L, P, V, W, Y; hydrophilic residues: C, D, E, H, K, M, N, Q, R, S, T); surface exposure groups including residues (exposed residues: D, E, H, K, N, P, Q, R, S, T, Y; buried residues: A, C, F, G, I, L, M, V, W); disorder groups including residues (disorder-promoting residues: A, R, S, Q, E, G, K, P; order-promoting residues: N, C, I, L, F, W, Y, V; disorder-order neutral residues: D, H, M, T); flexibility groups including residues (high flexibility residues: D, E, K, N, P, Q, R, S; low flexibility residues: A, C, F, G, H, I, L, M, T, V, W, Y).
- *Physicochemical properties* are encoded numerical values of properties for the residues in the window. For each physicochemical property, there is a set of 20 numerical values for amino acids. In our work, the top 6 physicochemical properties (see Table SS1) are selected and defined as informative features by comparing the prediction accuracy of each physicochemical property from AAindex . (total of 6×27 = 162 features).
- *KNN feature* generated by taking the local sequence around a possible modification site in a query protein and extracted features from its similar sequences in both positive and negative sets with a KNN algorithm as follows: For a query site (possible pupylation site), find its *k* nearest neighbors in positive and negative sets based on local sequence similarity, respectively. For two local sequence *s*_1_ and *s*_2_, the distance Dist(*s*_1_,*s*_2_) is defined as

where *p* denotes the number of flanking residues from the central site in the protein sequence fragment and *i* denotes the position of an amino acid in the target sequence segment; Sim, the amino acid similarity matrix, is derived from the BLOSUM62 substitution matrix as

$$\mathrm{Sim}\text{(}a\text{,}b\text{)}=\frac{\mathrm{M}(a\text{,}b)-\min\{M\}}{\max\{M\}-\min\{M\}}$$

where *a* and *b* are two amino acids, *M* is the substitution matrix, and max/min{*M*} represent the largest/smallest number in the matrix, respectively. The corresponding KNN feature is then extracted as follows: (1) Form a set of neighbors by combining the positive and negative sets, named Comparison set; (2) Calculate the average distances from the query sequences to Comparison set; (3) Sort the neighbors by the distances and pick the *k* nearest neighbors; (4) Calculate the KNN score, the percentage of positive neighbors (pupylation sites) in its *k* nearest neighbors; (5) In order to obtain multiple features, *k* was chosen to be different values (in this work *k*=0.5%, 1%, 2%, 4%, and 8% respectively) of the size of the training data set. In PupPred, five KNN scores were extracted as features for pupylation prediction. (total of 5 features).

- Predicted *secondary structure* generated by PSIPRED . We use “100”, “010” and “001” to encode the 3 secondary structure states (helix/strand/coil) for each residue in the window (total of 3×27 = 51 features).
- *PSSM profile* generated by PSIBLAST with default parameters using the Swiss-Prot non-redundant database using 3 iteration and e-value threshold for inclusion in multi-pass model 0.0001 (-h 0.0001). For each residue, there are 20 values indicating the probabilities of occurrences for 20 amino acids (total of 20×27 = 540 features).

**Table SS1**. **List the top six of the physicochemical properties that were selected as informative features by comparing the prediction accuracy of each physicochemical property.**

| **ID** | **Properties Description** | **Acc(%)** |
| --- | --- | --- |
| KARP850103 | Flexibility parameter for two rigid neighbors (Karplus-Schulz, 1985) | 60.766 |
| JANJ780103 | Percentage of exposed residues (Janin et al., 1978) | 60.683 |
| JANJ780101 | Average accessible surface area (Janin et al., 1978) | 60.267 |
| PONP800102 | Average gain in surrounding hydrophobicity (Ponnuswamy et al., 1980) | 59.906 |
| JANJ780101 | Average accessible surface area (Janin et al., 1978) | 59.750 |
| FAUJ880111 | Positive charge (Fauchere et al., 1988) | 59.567 |

**REFERENCES**

1. Chen Z, Chen YZ, Wang XF, Wang C, Yan RX, Zhang Z: **Prediction of ubiquitination sites by using the composition of k-spaced amino acid pairs**. *PLoS One* 2011, **6**(7):e22930.

2. Eisenberg D: **3-DIMENSIONAL STRUCTURE OF MEMBRANE AND SURFACE-PROTEINS**. *Annu Rev Biochem* 1984, **53**:595-623.

3. Janin J: **Surface and inside volumes in globular proteins**. *Nature* 1979, **277**(5696):491-492.

4. Dunker AK, Lawson JD, Brown CJ, Williams RM, Romero P, Oh JS, Oldfield CJ, Campen AM, Ratliff CR, Hipps KW *et al*: **Intrinsically disordered protein**. *J Mol Graph Model* 2001, **19**(1):26-59.

5. Vihinen M, Torkkila E, Riikonen P: **ACCURACY OF PROTEIN FLEXIBILITY PREDICTIONS**. *Proteins* 1994, **19**(2):141-149.

6. Kawashima S, Kanehisa M: **AAindex: Amino acid index database**. *Nucleic Acids Res* 2000, **28**(1):374-374.

7. Gao JJ, Thelen JJ, Dunker AK, Xu D: **Musite, a Tool for Global Prediction of General and Kinase-specific Phosphorylation Sites**. *Mol Cell Proteomics* 2010, **9**(12):2586-2600.

8. Henikoff S, Henikoff JG: **Amino-acid substitution matrices from protein blocks**. *P Natl Acad Sci Usa* 1992, **89**(22):10915-10919.

9. McGuffin LJ, Bryson K, Jones DT: **The PSIPRED protein structure prediction server**. *Bioinformatics* 2000, **16**(4):404-405.

10. Altschul SF, Madden TL, Schaffer AA, Zhang JH, Zhang Z, Miller W, Lipman DJ: **Gapped BLAST and PSI-BLAST: a new generation of protein database search programs**. *Nucleic Acids Res* 1997, **25**(17):3389-3402.
